# Impact of glucose metabolism on PD-L1 expression in sorafenib-resistant hepatocellular carcinoma cells

**DOI:** 10.1038/s41598-024-52160-x

**Published:** 2024-01-19

**Authors:** Sua Cho, Wonjin Kim, Dayoung Yoo, Yeonju Han, Hyemin Hwang, Seunghwan Kim, Jimin Kim, Sanghee Park, Yusun Park, HanHee Jo, Jae-chul Pyun, Misu Lee

**Affiliations:** 1 Division of Life Sciences, College of Life Science and Bioengineering, Incheon National University, Incheon, 22012 Republic of Korea; 2 Neurosciences Graduate Program, University of California, San Diego, La Jolla, CA USA; 3 Department of Materials Science and Engineering, Yonsei University, 50 Yonsei-Ro, Seodaemun-Gu, Seoul, 03722 Republic of Korea; 4 Institute for New Drug Development, College of Life Science and Bioengineering, Incheon National University, Incheon, 22012 Republic of Korea

**Keywords:** Cancer metabolism, Cancer therapy, Biochemistry, Cancer, Cell biology, Oncology

## Abstract

Hepatocellular carcinoma (HCC) is the fifth leading cause of cancer-related mortality worldwide. Programmed cell death ligand-1 (PD-L1) is an immune checkpoint protein that binds to programmed cell death-1 (PD-1), which is expressed in activated T cells and other immune cells and has been employed in cancer therapy, including HCC. Recently, PD-L1 overexpression has been documented in treatment-resistant cancer cells. Sorafenib is a multikinase inhibitor and the only FDA-approved treatment for advanced HCC. However, several patients exhibit resistance to sorafenib during treatment. This study aimed to assess the effect of glucose deprivation on PD-L1 expression in HCC cells. We used PD-L1-overexpressing HepG2 cells and IFN-γ-treated SK-Hep1 cells to explore the impact of glycolysis on PD-L1 expression. To validate the correlation between PD-L1 expression and glycolysis, we analyzed data from The Cancer Genome Atlas (TCGA) and used immunostaining for HCC tissue analysis. Furthermore, to modulate PD-L1 expression, we treated HepG2, SK-Hep1, and sorafenib-resistant SK-Hep1R cells with rapamycin. Here, we found that glucose deprivation reduced PD-L1 expression in HCC cells. Additionally, TCGA data and immunostaining analyses confirmed a positive correlation between the expression of hexokinase II (HK2), which plays a key role in glucose metabolism, and PD-L1. Notably, rapamycin treatment  decreased the expression of PD-L1 and HK2 in both high PD-L1-expressing HCC cells and sorafenib-resistant cells. Our results suggest that the modulation of PD-L1 expression by glucose deprivation may represent a strategy to overcome PD-L1 upregulation in patients with sorafenib-resistant HCC.

## Introduction

Hepatocellular carcinoma (HCC) is a leading cause of cancer-related mortality worldwide and the most common cause of death in individuals with cirrhosis^1^. Due to the asymptomatic nature of early-stage HCC, it is often only detected at intermediate or advanced stages, which reduces the effectiveness of curative treatments such as surgical resection, ablation, or liver transplantation^2^. Sorafenib (Nexavar®), an orally-active multikinase inhibitor, is the first-line chemotherapy for advanced HCC. Clinical studies have shown that sorafenib can significantly extend the median survival time of patients with advanced HCC by approximately 3–5 months^3^. However, its anticancer efficacy is hindered by the development of drug resistance in the cells, resulting in a poor prognosis for patients^4,5^. Therefore, it is important to restore susceptibility to sorafenib in patients with drug-resistant HCC.

Studies of the mechanisms underlying cancer immune evasion have focused on the programmed cell death protein 1 (PD-1)/programmed cell death ligand 1 (PD-L1) pathway. PD-L1, a member of the B7 protein family, interacts with PD-1, which is expressed on various immune cells, such as antigen-presenting cells and endothelial cells^6,7^. Activation of the PD-1/PD-L1 pathway leads to the suppression of immune cells and production of certain cytokines such as IFN-γ, resulting in the inhibition of the anti-tumor T cell response^8,9^. High expression of PD-1 and PD-L1 is associated with poor prognosis, increased HCC aggressiveness, and a higher risk of tumor relapse in patients who have undergone curative resection^10–13^. Nivolumab and pembrolizumab, two approved anti-PD-1 agents for HCC treatment, have exhibited durable responses in patients with advanced HCC who have received prior systemic therapy^14,15^. Pembrolizumab proved effective and well tolerated in patients with advanced HCC previously treated with sorafenib^15^. Notably, dysregulation of PD-L1 expression has been documented in several carcinomas following treatment with chemotherapeutic agents, including cisplatin, carboplatin, and paclitaxel^16,17^. In the case of HCC, a significant upregulation of PD-L1 expression was observed in HCC cells after sorafenib therapy^18,19^. Currently, a treatment approach to decrease sorafenib resistance-induced elevated PD-L1 expression is yet to be fully developed.

Cancer cells require increased energy, and to meet this high biosynthetic demand, they modify their metabolic flux via various metabolic pathways. In the 1920s, Otto Warburg was the first to observe cancer cells convert glucose into lactate, even in the presence of sufficient oxygen. This phenomenon, known as aerobic glycolysis or the Warburg effect, is primarily characterized by increased glucose uptake, enhanced glycolysis, restricted mitochondrial oxidative phosphorylation, and an upregulation of the pentose phosphate pathway^20^. The resulting aberrant accumulation of metabolites, produced through alterations in the metabolic pathways of HCC and other kinds of cancerous cells, modulates the microenvironment and induces resistance to chemotherapeutic agents, including sorafenib^21,22^.

Thus, this study aimed to assess the impact of glucose deprivation on PD-L1 expression in HCC cells and the correlation between PD-L1 expression and glycolysis in tumors of patients with HCC. Strategies to modulate PD-L1 expression in sorafenib-resistant HCC cells have also been explored.

## Results

### Low glucose concentrations are associated with PD-L1 expression downregulation

**Figure 1:**
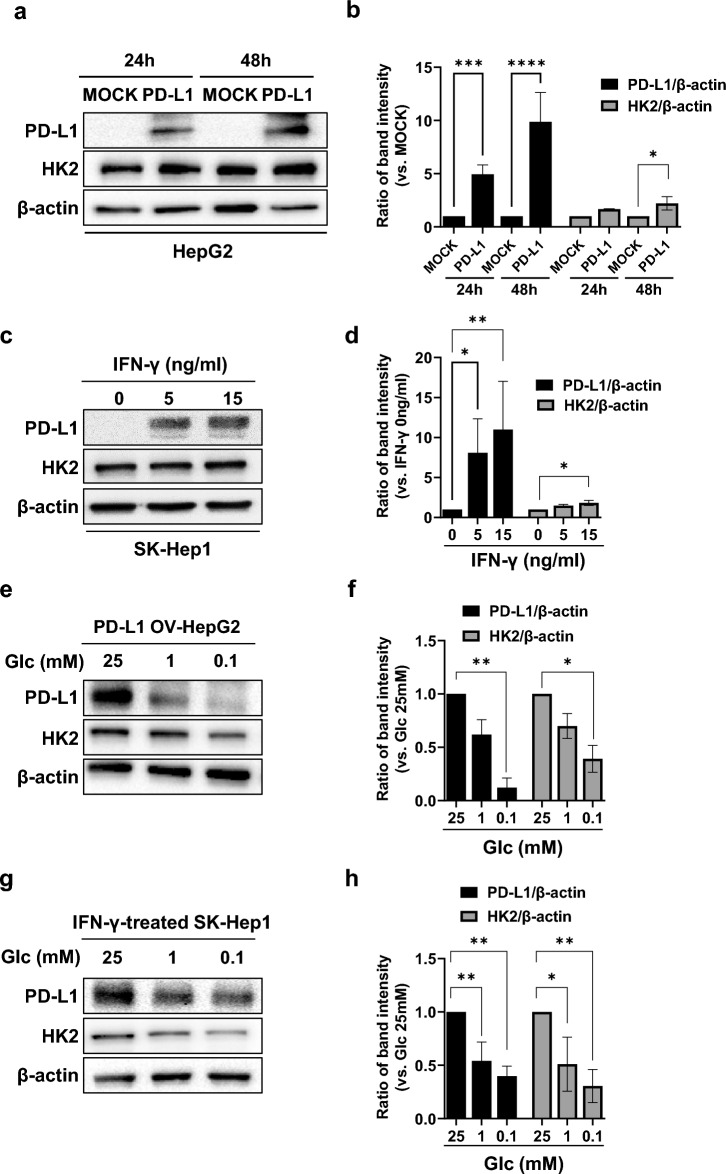
Reduced PD-L1 expression after glucose deprivation. (**a**) HepG2 cells were transfected with either MOCK or pCMV-PD-L1 plasmids. After the specified incubation time, PD-L1, HK2, and β-actin levels were evaluated using western blotting (WB). The full-length blots are in Supplementary Fig. S3. (**b**) Densitometry intensity ratios for (**a**) from replicated WB (n = 3). *; *p* < 0.05, ***; *p* < 0.001, ****; *p* < 0.0001. (**c**) SK-Hep1 cells were treated with the indicated concentration of IFN-γ. After 48 h, PD-L1, HK2, and β-actin levels were evaluated using WB. The full-length blots are shown in Supplementary Fig. S4. (**d**) Densitometry intensity ratios for (c) from replicated WB (n = 3). *; *p* < 0.05, **; *p* < 0.01. (**e**) HepG2 cells were transfected with pCMV-PD-L1 plasmid. After 48 h, the cells were treated with the indicated concentration of glucose for 8 h. PD-L1, HK2, and β-actin levels were evaluated using WB. The full-length blots are in Supplementary Fig. S5. (**f**) Densitometry intensity ratios for (**e**) from replicated WB (n = 3) analyses. *; *p* < 0.05, **; *p* < 0.01. (**g**) SK-Hep1 cells were treated with IFN-γ (15 ng/mL). After 48 h, the cells were treated with the indicated concentration of glucose for 8 h. PD-L1, HK2, and β-actin images were evaluated using WB. The full-length blots are in Supplementary Fig. S6. (**h**) Densitometry intensity ratios for (**g**) from replicated WB analyses (n = 3). *; *p* < 0.05, **; *p* < 0.01.

The expression levels of PD-L1 were evaluated in four human HCC cell lines after treatment with IFN-γ. Following treatment, PD-L1 was markedly upregulated in the SK-Hep1 and Hep3B cell lines, but not in the Hep2 and Huh7 cell lines (Supplementary Fig. S1). To investigate the effects of glycolysis on PD-L1 expression, we used PD-L1 overexpressed HepG2 (low PD-L1 expression) and IFN-γ-treated-SK-Hep1 (high PD-L1 expression) cells. The expression levels of PD-L1 and HK2, hallmarks of glycolysis, were increased in PD-L1-overexpressed HepG2 cells (Fig. 1a,b) and IFN-γ-treated SK-Hep1 cells (Fig. 1c,d). Elevated PD-L1 and HK2 expression levels decreased after glucose deprivation both in PD-L1-overexpressed HepG2 cells (Fig. 1e,f) and IFN-γ-treated SK-Hep1 cells (Fig. 1g,h). These findings suggest a potential association between PD-L1 expression and glycolysis.

### Correlation between PD-L1 expression and glycolysis in patients with HCC

**Figure 2:**
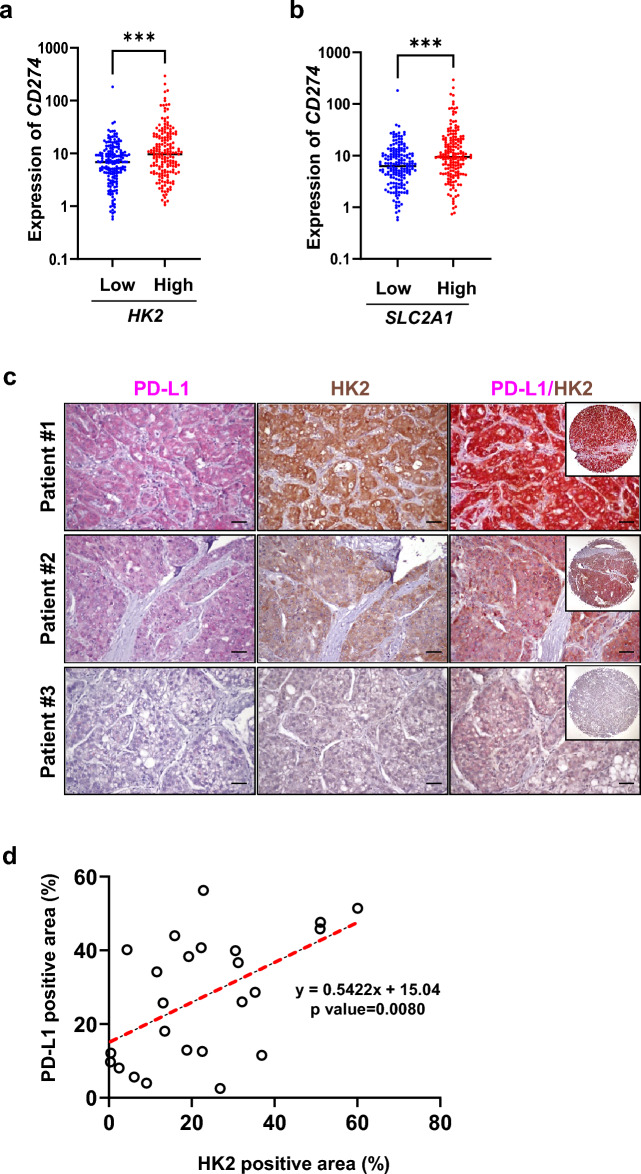
Positive correlation between PD-L1 and HK2 in patients with HCC. (**a**) *CD274* expression with low and high *HK2* expression groups (50–75 percentile) in the Cancer Genome Atlas (TCGA) dataset of patients with HCC (n = 360). ***; *p* < 0.001. (**b**) *CD274* expression with low and high *SLC2A1* expression groups (50–75 percentile) in the TCGA dataset of patients with HCC (n = 360). ***; *p* < 0.001. (**c**) Immunohistochemistry was performed on a tissue microarray (with 24 patients) using PD-L1 (magenta pink) or HK2 (brown) antibodies. Co-staining was performed with both PD-L1 and HK2. Counterstaining was performed with hematoxylin. Scale bars: 50 μm. (**d**) Correlation between the PD-L1-positive area and HK2-positive area. Pearson’s correlation coefficient was used to calculate the correlation between the two factors.

The Cancer Genome Atlas (TCGA), using the OncoLnc TCGA data portal (www.oncolnc.org), and immunostaining assays on HCC tissues were used to establish a correlation between PD-L1 expression and glycolysis. TCGA analysis revealed a significant positive correlation between elevated *HK2* expression and *CD274* expression in patients with HCC (n = 360, Fig. 2a). Additionally, a similar association was observed with elevated *SLC2A1* expression, which encodes glucose transporter protein type 1 (GLUT1; Fig. 2b). Further, the protein expression levels of PD-L1 and HK2 were verified using an HCC tissue microarray comprising 24 samples (Fig. 2c and Supplementary Table S1). A statistically significant positive correlation was observed between the expression levels of PD-L1 and HK2 (R^2^ = 0.3878, *p* = 0.0080; Fig. 2d). However, the limited sample size precluded a statistically significant correlation from being established between PD-L1 and the TNM Classification of Malignant Tumors (TNM) stages. Overall, these findings confirm a positive correlation between PD-L1 expression and glycolysis in patients with HCC.

### Upregulation of PD-L1 expression in sorafenib-resistant HCC cells

**Figure 3:**
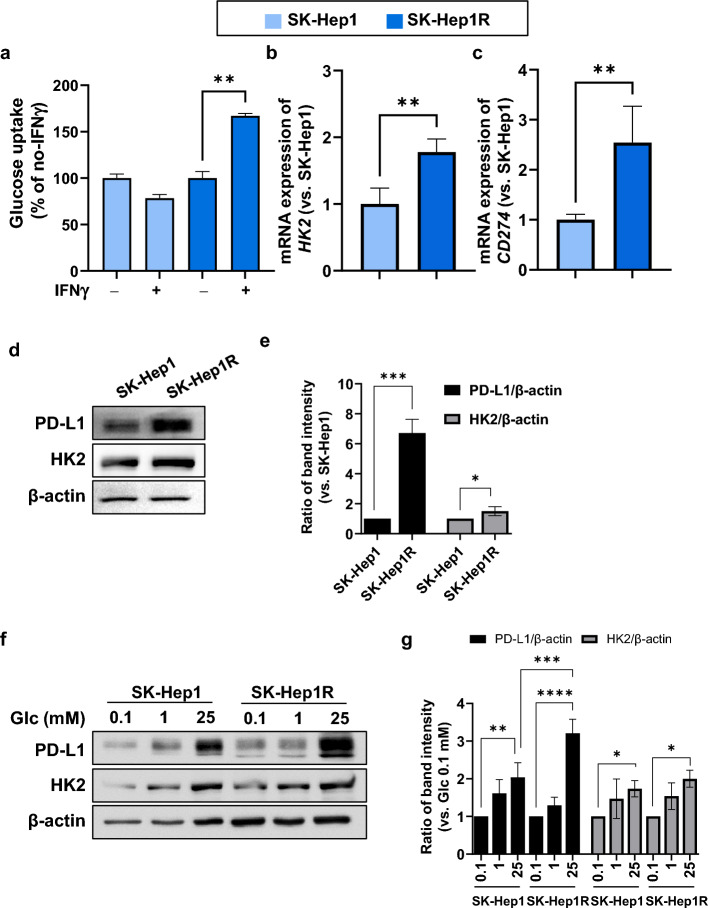
Upregulation of PD-L1 in sorafenib-resistant HCC cells. (**a**) SK-Hep1 and SK-Hep1R cells were treated with or without IFN-γ (15 ng/mL). After 48 h, glucose uptake was measured. Data are shown as the mean of three independent experiments ± SD. **; *p* < 0.01 (**b**,**c**) SK-Hep1 and SK-Hep1R cells were treated with IFN-γ (15 ng/mL). After 48 h treatment, the expression levels of *HK2* (**b**) and *CD274* (**c**) were analyzed by quantitative real-time PCR. The expression levels of target genes were normalized to that of the housekeeping gene *GAPDH* based on the 2^−ΔΔCt^ method. Data are shown as the mean of three independent experiments ± SD. **; *p* < 0.01. (**d**) SK-Hep1 and SK-Hep1R cells were treated with IFN-γ (15 ng/mL) for 48 h. PD-L1, HK2, and β-actin protein levels were evaluated by WB. The full-length blots are in Supplementary Fig. S7. (**e**) Densitometry intensity ratios for (**d**) from replicated WB analyses (n = 3). *; *p* < 0.05, ***; *p* < 0.001. (**f**) SK-Hep1 and SK-Hep1R cells were treated with IFN-γ (15 ng/mL). After 48 h, the cells were treated with the indicated concentration of glucose for 8 h. PD-L1, HK2, and β-actin protein levels were evaluated using WB. The full-length blots are shown in Supplementary Fig. S8. (**g**) Densitometry intensity ratios for (**f**) from replicated WB analyses (n = 3). *; *p* < 0.05, **; *p* < 0.01, ***; *p* < 0.001, ****; *p* < 0.0001.

Sorafenib is a commonly used first-line chemotherapy for the treatment of advanced HCC; however, patients often develop drug resistance within 6 months^3^. To comprehend the correlation between PD-L1 expression, glycolysis, and sorafenib resistance, we employed sorafenib-resistant SK-Hep1 cells, which are induced through exposure to increasing concentrations of sorafenib (1–10 μM) over 6 months. (IC_50_ of SK-Hep1 = 9.1 μM, IC_50_ of SK-Hep1R = 18.9 μM, Supplementary Fig. S2). Glucose uptake increased in SK-Hep1R cells following IFN-γ treatment compared to that in corresponding SK-Hep1 cells (Fig. 3a), as well as the upregulation of *HK2* expression (Fig. 3b). The expression of *CD274* was upregulated in SK-Hep1R cells compared with that in SK-Hep1 cells (Fig. 3c). Similar to the RNA expression levels, PD-L1 in SK-Hep1R cells exhibited much higher expression than the SK-Hep1 cells at the protein level, while HK2 showed a marginal increase in SK-Hep1R cells (Fig. 3d,e). Furthermore, glucose treatment resulted in a significant elevation of PD-L1 levels in SK-Hep1R cells compared to SK-Hep1 cells (Fig. 3f,g). These results suggest that glucose deprivation is a potential strategy for reducing elevated PD-L1 expression in highly glycolytic, sorafenib-resistant HCC cells.

### Regulation of PD-L1 expression following rapamycin treatment

**Figure 4:**
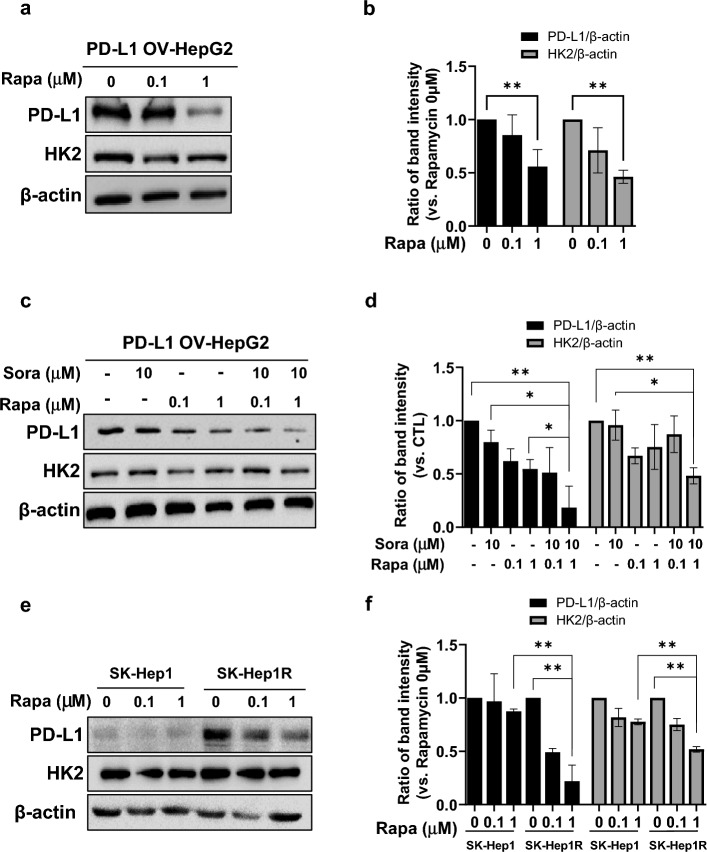
Downregulation of PD-L1 expression after rapamycin treatment. (**a**) HepG2 cells were transfected with pCMV-PD-L1. After 48 h, the cells were incubated with rapamycin for an additional 24 h. The full-length blots are in Supplementary Fig. S9. (**b**) Densitometry intensity ratios for (a) from replicated western blots (WBs) (n = 3). **; *p* < 0.01. (**c**) HepG2 cells were transfected with pCMV-PD-L1. After 24 h, the cells were incubated for an additional 24 h with/without rapamycin and/or sorafenib at indicated concentrations. PD-L1, HK2, and β-actin levels were evaluated through WB. The full-length blots are in Supplementary Fig. S10. (**d**) Densitometry intensity ratios for (**c**) from replicated WB analyses (n = 3) *; *p* < 0.05, **; *p* < 0.01. (**e**) SK-Hep1 and SK-Hep1R cells were treated with IFN-γ (15 ng/mL). After 48 h, the cells were incubated for an additional 6 h with/without rapamycin at indicated concentrations. PD-L1, HK2, and β-actin levels were evaluated using WB. The full-length blots are shown in Supplementary Fig S11. (**f**) Densitometry intensity ratios for (e) from replicated WB (n = 3) **; *p* < 0.01.

Autophagy induction has been observed following glucose deprivation in cells^23^. Therefore, to regulate PD-L1 expression, rapamycin, an autophagy inducer utilized in several clinical trials for treating advanced HCC, was employed. Rapamycin treatment induced a reduction in PD-L1 and HK2 expression levels in HepG2 cells overexpressing PD-L1, similar to the effects of glucose deprivation (Fig. 4a,b). Notably, combined treatment with rapamycin (0.1 µM and 1 µM) and sorafenib (10 µM) resulted in the lowest PD-L1 expression in HepG2 cells with PD-L1 overexpression, compared to single treatments (Fig. 4c,d). The expression of HK2 after combination treatment showed levels lower than those observed with single sorafenib treatment, but not lower than rapamycin treatment. SK-Hep1R cells, treated with IFN-γ, also exhibited reduced PD-L1 and HK2 expression after rapamycin treatment. SK-Hep1R cells demonstrated a more pronounced reduction in PD-L1 and HK2 compared to SK-Hep1 cells (Fig. 4e,f). Thus, rapamycin treatment might regulate PD-L1, which is amplified in sorafenib-resistant cells.

### Enhanced anticancer activity following rapamycin treatment

**Figure 5:**
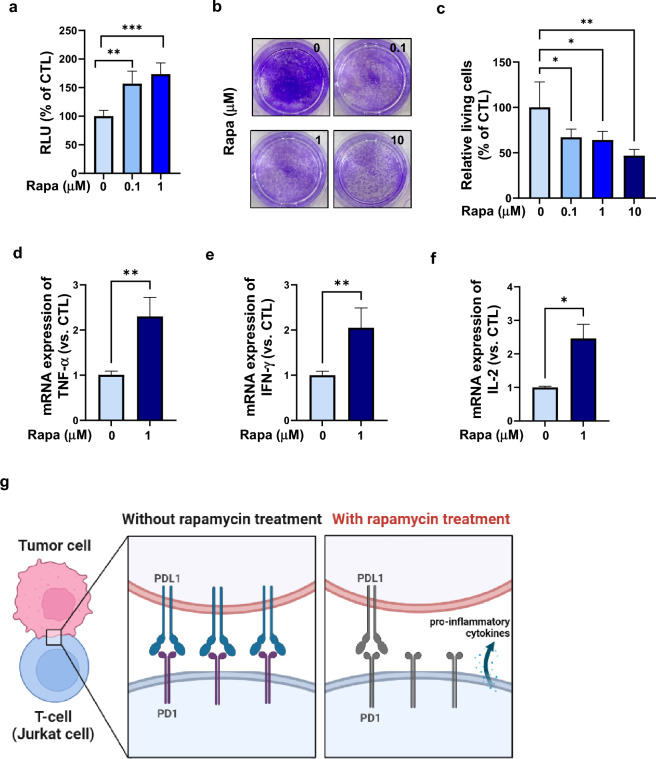
Enhanced anticancer effect following rapamycin treatment. (**a**) PD-L1 and PD-1 binding affinity following rapamycin treatment were measured by co-culture experiments performed using Jurkat-Lucia™ TCR-hPD-1 cells (1 × 10^5^ cells/mL) and Raji-APC-hPD-L1 cells (5 × 10^4^ cells/mL). Relative light units (RLUs) were measured using a luminometer. **; *p* < 0.01, ***; *p* < 0.001. (**b**) SK-Hep1R cells were plated. The following day, Jurkat-Lucia™ TCR-hPD-1 cells were added with rapamycin and incubated for 48 h. After washing with phosphate-buffered saline (PBS) thrice, the cells were fixed with methanol and stained with crystal violet, and images were recorded. Magnification: ×40. (**c**) Quantification of positive cells of (**b**). *; *p* < 0.05, **; *p* < 0.01. (**d**,**e**,**f**) Total RNA was extracted from Jurkat-Lucia™ TCR-hPD-1 cells after 48 h of co-culture with SK-Hep1R cells with or without rapamycin, and cDNA was synthesized. Quantitative real-time PCR was performed to analyze the expression levels of *TNF-α* (**d**), *IFN-γ* (**e**), and *IL-2* (**f**). Target gene expression was normalized to that of the housekeeping gene *GAPDH* using the 2^-ΔΔCt^ method. Data represent the mean of three independent experiments ± SD. *; *p* < 0.05, **; *p* < 0.01. (**g**) Schematic diagram illustrating the co-culture system of SK-Hep1R HCC cells and PD-1 Jurkat cells. The diagram shows the SK-Hep1R cells (in red) expressing PD-L1 and Jurkat cells expressing PD-1 (in blue). Following rapamycin treatment, PD-L1 expression was decreased and pro-inflammatory cytokines were released from Jurkat cells expressing PD-1.

To evaluate the PD-L1 and PD-1 binding affinity following rapamycin treatment, co-culture experiments were performed using Jurkat-Lucia™ TCR-hPD-1 and Raji-APC-hPD-L1 cells. Jurkat-Lucia™ TCR-hPD-1 cells were genetically engineered to produce a bioluminescent signal when the interaction between PD-L1 and PD-1 is inhibited. Relative light units (RLUs) increased after rapamycin treatment, indicating that rapamycin decreased the binding affinity between PD-L1 and PD-1 in the co-culture system (Fig. 5a). Since rapamycin reduced the binding of PD-1 and PD-L1, the cytolytic activity of Jurkat-Lucia™ TCR-hPD-1 cells was measured in co-culture with SK-Hep1R cells to examine the potential anticancer effect. Rapamycin enhanced the cytolytic activity of Jurkat-Lucia™ TCR-hPD-1 cells in these co-cultures (Fig. 5b,c). Compared with the control, the mRNA expression levels of the pro-inflammatory cytokines TNF-α, IFN-γ, and IL-2 were significantly increased in Jurkat-Lucia™ TCR-hPD-1 cells after co-culture with SK-Hep1R cells treated with rapamycin (Fig. 5d–f). In conclusion, rapamycin caused a reduction in PD-L1 levels and significant induction of T cell-mediated cytotoxicity against SK-Hep1R cells (Fig. 5g).

## Discussion

Increased glycolysis has been linked to chemotherapy resistance and poor clinical outcomes in patients with HCC^24,25^. Recent studies have supported the notion that elevated PD-L1 expression is associated with increased glycolysis in tumors, leading to the immune escape of tumor cells^26^. In the present study, we confirmed the positive correlation between PD-L1 expression and glycolysis in sorafenib-resistant HCC cells. We also established that rapamycin treatment enhances the cytolytic activity of immune cells by decreasing PD-L1 expression in sorafenib-resistant HCC cells.

Sorafenib has been shown to improve survival outcomes in patients with HCC; however, its long-term efficacy is impaired by the emergence of resistance through various mechanisms. A recent study by Lu et al. identified elevated PD-L1 expression in tumor-infiltrating immune cells of patients with HCC following sorafenib treatment^18^. Our study further revealed the upregulation of *CD274*/PD-L1 in HCC cells upon acquisition of sorafenib resistance. The upregulation of PD-L1 aggravates sorafenib-resistant HCC cells by promoting epithelial-mesenchymal transition through the PI3K/Akt pathway^27^. Liu et al. also demonstrated that the PD-L1/DNMT1 axis plays a role in sorafenib resistance in HCC, and inhibiting both PD-L1 and DNMT1 expression could restore the sensitivity of cells to sorafenib^28^. These previous studies lacked a clear understanding of why PD-L1 expression is increased in sorafenib-resistant cells. Our study revealed that sorafenib-resistant cells exhibit high glucose uptake and that this increase in glycolysis leads to elevated PD-L1 levels. Hypoxia, a major mechanism underlying glycolysis and cancer therapy resistance, induces PD-L1 upregulation in various tumor cells^29,30^. Therefore, hypoxia-induced glycolysis may contribute to the upregulation of PD-L1 and HK2 in patients with HCC. Hypoxia has been reported to be a major driver of resistance to cancer therapeutics^31^.

The FDA has approved the use of rapamycin for renal and liver transplantation. Patients with recurrent HCC display high levels of PD-L1 expression and reduced relapse-free survival compared with those with lower PD-L1 expression^19^. Recent studies have demonstrated a reduction in PD-L1 expression in non-small cell lung cancer lines following exposure to rapamycin, an autophagy inducer^32^. Our study shows that rapamycin treatment reduces PD-L1 expression, making it more effective against sorafenib-resistant cells. The interplay between autophagy and the PD-1/PD-L1 axis has been studied in various cancers. Wang et al. discovered that autophagy regulates PD-L1 expression in gastric cancer via the p62/SQSTM1-NF-κB pathway^33^. Further, in bladder cancer, PD-L1 mRNA stability, and expression are regulated by the ATG7/autophagy/FOXO3A/miR-145 axis^34^. Recent studies have reported the synergistic anticancer effects of a combination of sorafenib and rapamycin treatment on HCC cells. Gulhati et al. demonstrated that the addition of sorafenib to rapamycin treatment resulted in the abrogation of rapamycin-induced activation of the PI3K/Akt and Ras-MAPK signaling pathways, leading to enhanced antitumor effects^5^. Combination therapy significantly reduced tumor cell proliferation and increased the suppression of tumor cell angiogenesis compared to a single treatment in an in vivo animal model^35^. Additionally, our study highlights the potential anticancer effects of combining rapamycin therapy with sorafenib in HCC, with specific emphasis on the treatment of sorafenib-resistant HCC. Long-term treatment with other anticancer drugs has been shown to alter PD-L1 expression. High levels of PD-L1 have been observed in cisplatin-resistant small-cell lung carcinoma cells and enzalutamide-resistant prostate cancer cells^36^, suggesting the need for further investigation into the effects of rapamycin and other anticancer drugs on resistant cancer cells.

The overexpression of PD-L1 in various tumors is associated with patient survival and tumor recurrence. PD-L1 expression is a significant prognostic indicator of overall survival in HCC^37^. Additionally, PD-L1 regulates stem-like properties and contributes to tumor invasion. Thus, reducing PD-L1 expression, which is associated with tumorigenesis, is crucial for effective tumor treatment.

## Conclusions

Our findings suggest that sorafenib resistance leads to increased PD-L1 expression through elevated glucose metabolism, resulting in decreased sensitivity to PD-1/PD-L1 inhibitor therapy. Collectively, the combination of rapamycin and sorafenib presents a new therapeutic option for counteracting the upregulation of PD-L1 expression in sorafenib-resistant HCC cells, leading to a reduction in tumor aggressiveness.

## Methods

### Chemicals

Sorafenib (Santa Cruz Biotechnology, Dallas, TX, USA), rapamycin (Selleckchem, Bristol, UK and Thermo Fisher Scientific, Waltham, MA, USA), IFN-γ (Gibco, Grand Island, New York) and D-(+)-glucose (Sigma Aldrich, St. Louis, MO, USA) were used in this study. Sorafenib and rapamycin were dissolved in dimethyl sulfoxide (DMSO; Sigma Aldrich) and stored at − 80 °C. IFN-γ was dissolved in distilled water at a concentration of 10 mM and stored at − 80 °C. D-( +)-glucose was dissolved in distilled water at a concentration of 1 M and stored at 4 °C.

### Cell culture and generation of SK-Hep1R cells

Human HCC cell lines—HepG2, Hep3B, SK-Hep1, and Huh7 were purchased from the Korean Cell Line Bank (Seoul, Korea); Jurkat-Lucia^TM^ TCR-hPD-1 and Raji-APC-hPD-L1 cells were purchased from InvivoGen (San Diego, CA, USA). HepG2 cells were cultured in RPMI-1640 (Thermo Fisher Scientific), whereas Hep3B, SK-Hep1, and Huh7 were cultured in Dulbecco's Modified Eagle's Medium (DMEM; Thermo Fisher Scientific). Jurkat-Lucia™ TCR-hPD-1 and Raji-APC-hPD-L1 cells were cultured in modified Iscove’s Modified Dulbecco’s Medium (IMDM; Thermo Fisher Scientific) with selective antibiotics and handled following the manufacturer's recommendations. All media were supplemented with 10% heat-inactivated fetal bovine serum (HyClone, Logan, UT, USA) and 1% penicillin–streptomycin. Cells were maintained in a humidified incubator (Thermo Fisher Scientific) with 5% CO_2_ at 37 °C. SK-Hep1 cells were first continuously exposed to increasing concentrations of sorafenib (1–10 μM) over 6 months. Sorafenib resistance was examined by cell viability assay. Sorafenib-resistant SK-Hep1 (SK-Hep1R) cells exhibited a diminished reduction in cell proliferation after sorafenib treatment as compared to that with SK-Hep1 cells. The 50% inhibitory concentration (IC_50_) was calculated by performing a nonlinear regression analysis using GraphPad Prism (Supplementary Figure S2, IC_50_ of SK-Hep1 = 9.1 μM, IC_50_ of SK-Hep1R = 18.9 μM).

### Cell viability and glucose uptake assays

A Cell Counting Kit-8 (CCK-8, DOJINDO Laboratories, Kumamoto, Japan) assay was performed according to the manufacturer’s recommendations. To measure the binding affinity of PD-L1 for PD-1, Raji-APC-hPD-L1 cells (approximately 5 × 10^4^ cells/mL) were pre-incubated with rapamycin for 6 h and co-cultured with Jurkat-Lucia™ TCR-hPD-1 cells (approximately 1 × 10^5^ cells/mL) in a 96-well plate. After an additional 24 h of incubation, 50 µL of QUANTI‑Luc™ 4 reagent (InvivoGen) was added to each well, and luminescence was immediately measured using a luminescence microplate reader (PerkinElmer, Waltham, Massachusetts, USA). To measure cytolytic activity, SK-Hep1R cells were maintained for 48 h at 37 °C in 12-well plates at an effector cell: target cell (E:T) ratio of 10:1. The cytolytic activity was assessed using crystal violet staining (0.25% v/v in phosphate-buffered saline (PBS)) after fixation with methanol. Positive tumor regions were quantified using cellSens Imaging software (Olympus, Tokyo, Japan), with images acquired using a BX53 light microscope (Olympus). Glucose uptake was determined using a Glucose Assay kit (Promega, Madison, Wisconsin, USA) according to the manufacturer's instructions. Absorbance was measured at 440 and 640 nm using a microplate reader (Molecular Devices, San Jose, CA, USA).

### Real-time (RT) PCR and TCGA data

Total RNA was extracted using an RNeasy Mini Kit (Qiagen, Hilden, Germany). cDNA was synthesized from 500 ng of total RNA using the ReverTra Ace qPCR RT Master Mix with gDNA Remover (Toyobo, Osaka, Japan). Quantitative RT-PCR was performed using a C1000 Thermal Cycler (Bio-Rad Laboratories, Hercules, CA, USA) with SYBR Green RT PCR Master Mix (Toyobo). Gene expression levels were normalized to those of *GAPDH* mRNA in the corresponding cDNA samples. The following primers were used for this assay: *CD274* (forward, 5′-AAGAAAAGGGAGCACACAGG-3′ and reverse, 5′-GCCCAAGATGACAGACGATG-3′), *HK2* (forward 5′-AAGGTAGAAATGGAGCGAGGT-3′, reverse 5′-CCCGGAAATTTGTTCCTCCAA-3′), *hTNF-α* (forward 5′-TTCTCCTTCCTGATCGTGGCA-3′, reverse 5′-TAGAGAGAGGTCCCTGGGGAA-3′), *hIFN-γ* (forward 5′-TCGGTAACTGACTTGAATGTCCA-3′, reverse 5′-TCGCTTCCCTGTTTTAGCTGC-3′), *hIL-2* (forward 5′-AAGAATCCCAAACTCACCAG-3′, reverse 5′-CGTTGATATTGCTGATTAAGTCC-3′), and *hGAPDH* (forward, 5′-ACAGTCAGCCGCATCTTCTT-3′ and reverse 5′-TTGATTTTGGAGGGATCTCG-3′). For TCGA analysis, a set of 360 HCC samples, including high- and low-gene expression groups (50–75 percentile), was obtained from the OncoLnc TCGA data portal (www.oncolnc.org).

### Western blotting

Western blotting (WB) was performed as described previously^38^. HepG2 cells were transfected with pCMV-PD-L1 (Sinobio, Wayne, PA, USA), and SK-Hep1/SK-Hep1R cells were treated with IFN-γ. After incubation for 48 h, proteins were extracted using lysis buffer. Protein concentrations were measured and validated using a Nanodrop spectrophotometer (DeNovix Inc, Wilmington, USA), and 20 µg of protein was used for WB. Primary antibodies used in the present study were anti-PD-L1 (catalog no. 29122, Cell Signaling Technology, Danvers, MA, USA 1:500), anti-HKII (catalog no. ab209847; Abcam, Cambridge, UK; 1:500), and anti-β-actin (Santa Cruz Biotechnology, catalog no. sc-47778; 1:5000). WB using biological replicates showed similar expression data, indicating the reproducibility of the results. For band quantification, images were analyzed using ImageJ software^39^.

### Immunostaining

The tissue microarray, which contained samples from 24 cases of HCC, was purchased from TissueArray.Com LLC (catalog no. LV246, Derwood, MD, USA). Immunohistochemistry (IHC) was performed using an ImmPRESS Duet Double Staining Polymer Kit (HRP Anti-Mouse IgG-brown, AP Anti-Rabbit IgG-magenta; Vector Laboratories, Burlingame, CA, USA). Following antigen retrieval, immunostaining was performed with various antibodies. The primary antibodies used were anti-PD-L1 XP (Cell Signaling Technology; catalog no. 13684; 1:150) and anti-HKII (Abcam; catalog no. ab104826; 1:300). Images were recorded using an Olympus BX53 microscope and analyzed using Olympus CellSens software.

### Statistical analysis

Statistical analyses were performed using GraphPad Prism software (GraphPad Software Inc., San Diego, CA, USA). One-way analysis of variance (ANOVA) with Tukey's multiple comparison tests was performed to detect differences between three or more groups. A paired two-tailed Student’s *t*-test was used to detect significant differences between the two sets of data, and *p* < 0.05 was considered statistically significant.

### Supplementary Information

Supplementary Information.

## Data Availability

All data generated or analyzed during this study are included in this published article and its supplementary information files.
