# Supplementary material for: Impact of glucose metabolism on PD-L1 expression in sorafenib-resistant hepatocellular carcinoma cells
Source: Sci Rep. 2024 Jan 19;14:1751. doi: 10.1038/s41598-024-52160-x (PMC10798953; doi:10.1038/s41598-024-52160-x)
Supplement: Supplementary file 1 — Supplementary Information. [file 41598_2024_52160_MOESM1_ESM.docx]

**Supplementary information**

**Impact of glucose metabolism on PD-L1 expression in sorafenib-resistant hepatocellular carcinoma cells**

Sua Cho^1,†^, WonJin Kim^1,†^, Dayoung Yoo^1^, Yeonju Han^1^, Hyemin Hwang^1^, Seunghwan Kim^1^, Jimin Kim^1^, Sanghee Park^1^,Yusun Park^1^, HanHee Jo^1^, Jae-chul Pyun^3^, Misu Lee^1,2,*^

^1^Division of Life Sciences, College of Life Science and Bioengineering, Incheon National University, Incheon, 22012, South Korea

^2^Institute for New Drug Development, College of Life Science and Bioengineering, Incheon National University, 22012, South Korea.

^3^Department of Materials Science and Engineering, Yonsei University, 50 Yonsei-Ro, Seodaemun-Gu, Seoul 03722, Republic of Korea.

† These authors equally contributed to this study.

* **Corresponding authors**:

Misu Lee, PhD

Incheon National University, Incheon, Republic of Korea

E-mail: misulee@inu.ac.kr

Tel.: +82 32 835 8091

Fax: + 8232 835 0754


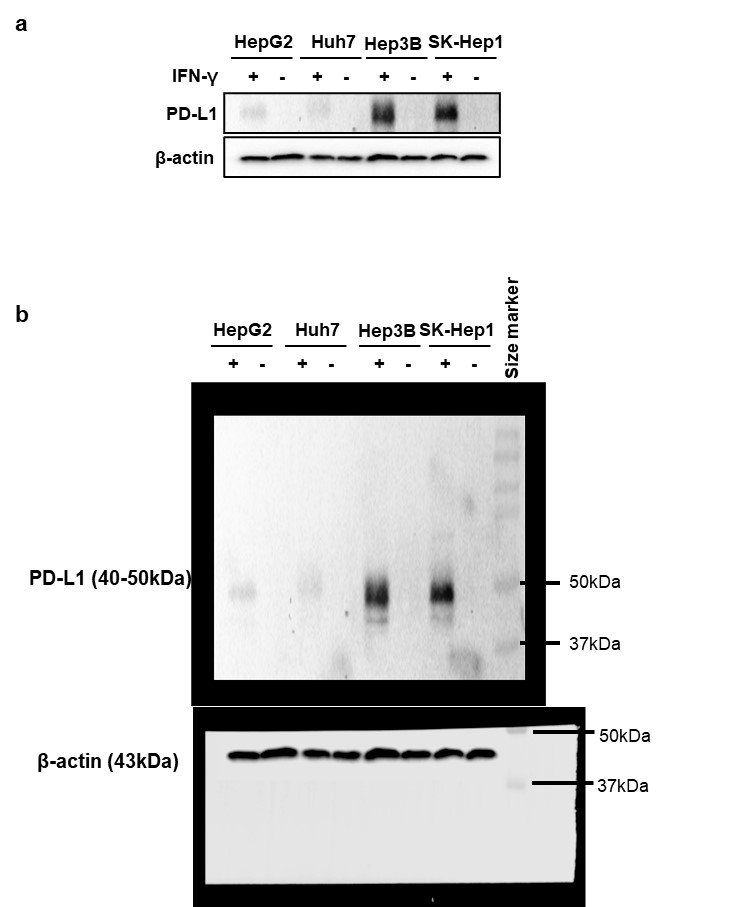


**Supplementary Figure S1. Expression of PD-L1 after IFN-γ treatment.** (a) HCC cells (HepG2, Huh7, Hep3B, and SK-Hep1 cells) were treated with the indicated concentration of IFN-γ. After 48 h, the expression of PD-L1 and β-actin were evaluated by western blotting. (b) Whole blot imaging of (a).


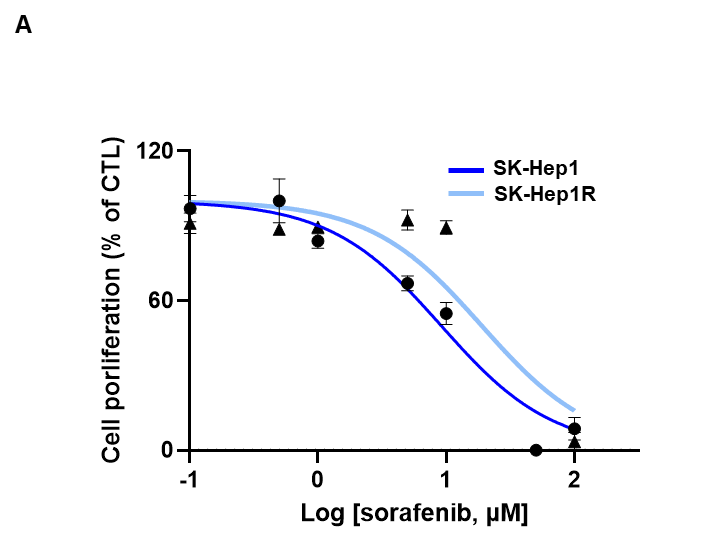


**Supplementary Figure S2. Cell viability of SK-Hep1 and SK-Hep1R after treatment with sorafenib.** Cell proliferation of SK-Hep1 and SK-Hep1R after treatment with the indicated concentration of sorafenib for 48 h. Data are shown as the mean of three independent experiments ± SD.


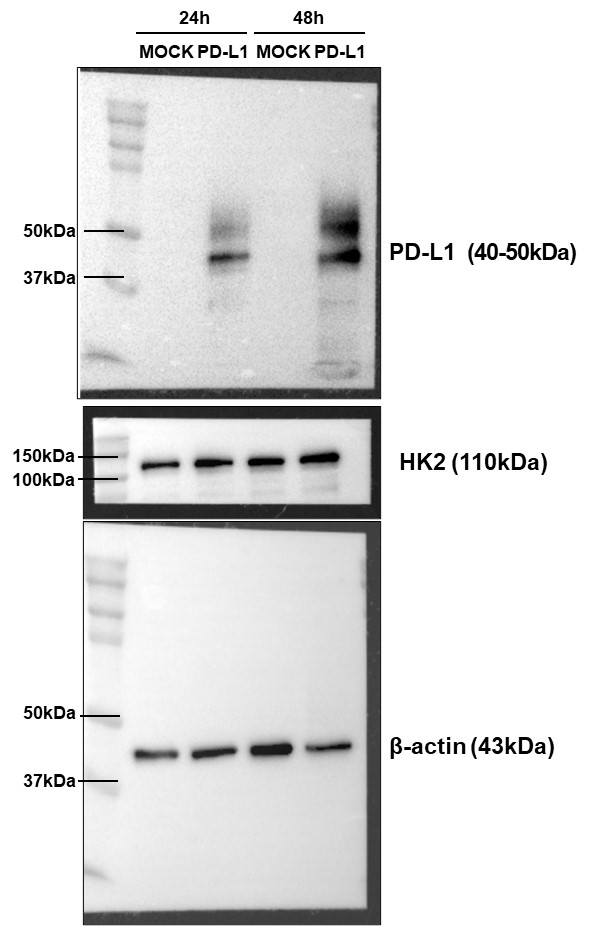


**Supplementary Figure S3.** Whole blot images of Fig 1a. The PD-L1 and β-actin images are from the same blot, while the HK2 images are from an upper blot of the PD-L1.

**
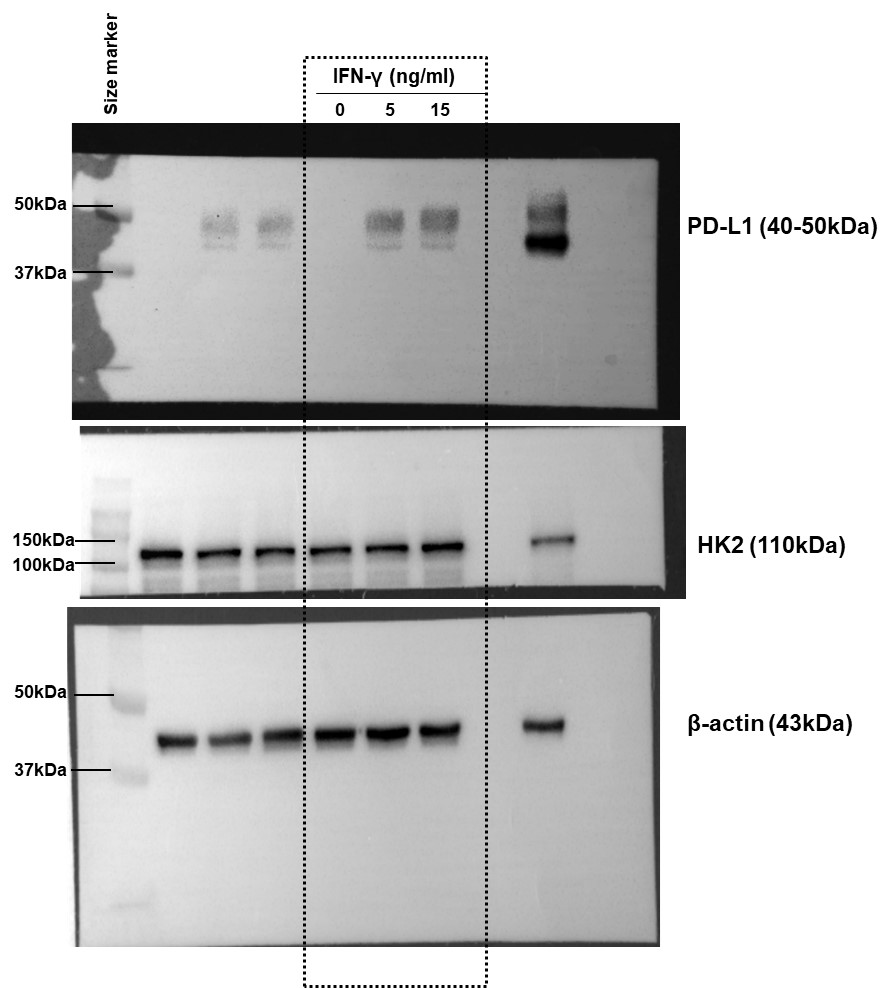
**

**Supplementary Figure S4.** Whole blot images of Fig 1c. The PD-L1 and β-actin images are from the same blot, while the HK2 images are from an upper blot of the PD-L1.


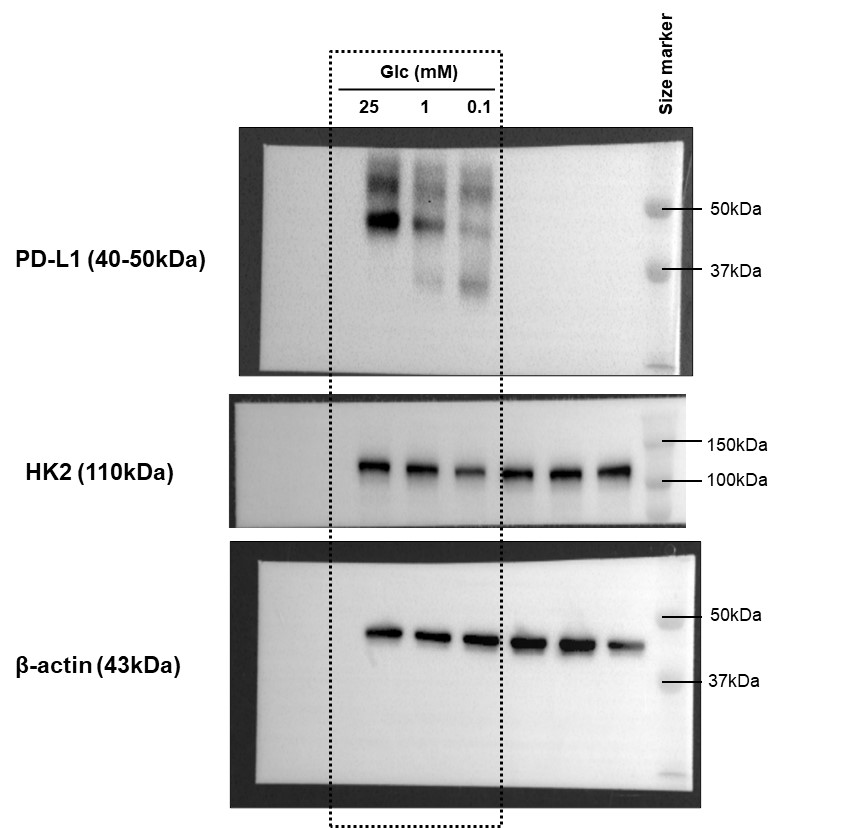


**Supplementary Figure S5.** Whole blot images of Fig 1e. The PD-L1 and β-actin images are from the same blot, while the HK2 images are from an upper blot of the PD-L1.


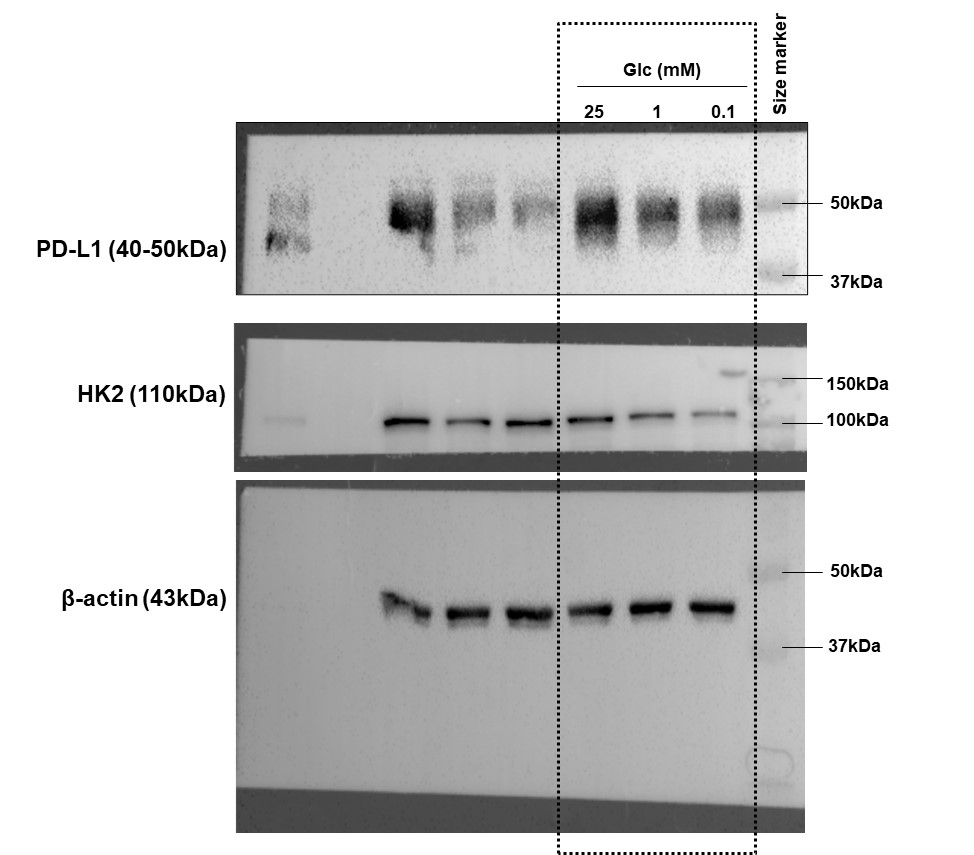


**Supplementary Figure S6.** Whole blot images of Fig 1g. The PD-L1 and β-actin images are from the same blot, while the HK2 images are from an upper blot of the PD-L1.


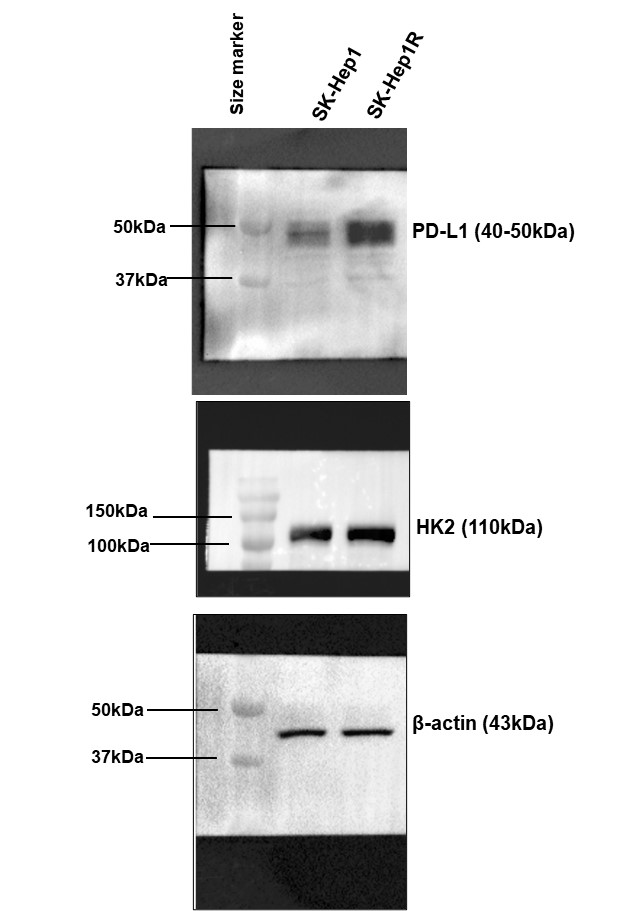


**Supplementary Figure S7.** Whole blot images of Fig 3d. The PD-L1 and β-actin images are from the same blot, while the HK2 images are from an upper blot of the PD-L1.

s


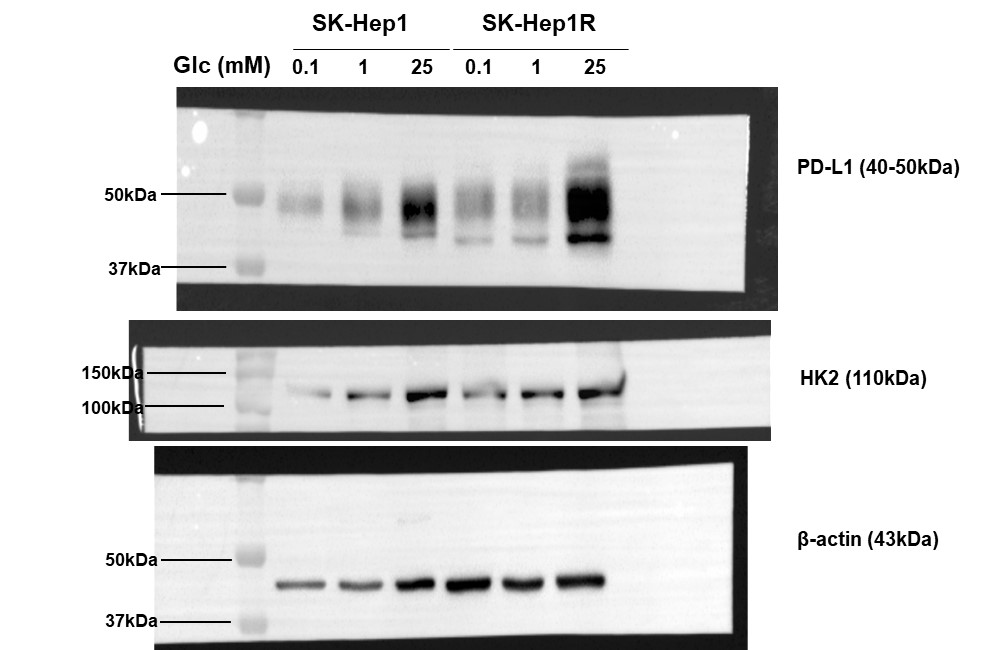


**Supplementary Figure S8.** Whole blot images of Fig 3f. The PD-L1 and β-actin images are from the same blot, while the HK2 images are from an upper blot of the PD-L1.

s


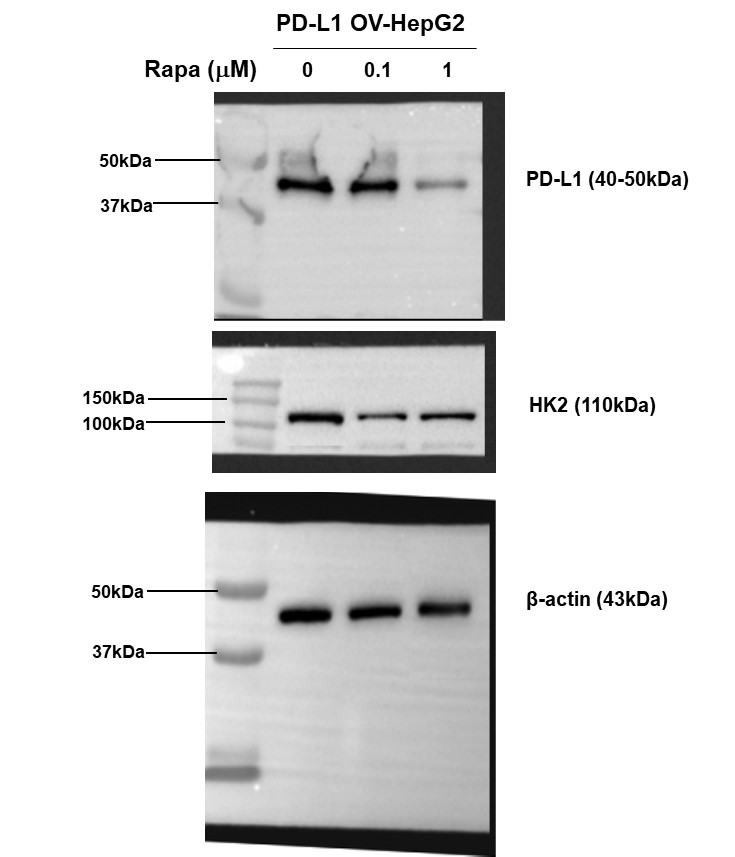


**Supplementary Figure S9.** Whole blot images of Fig 4a. The PD-L1 and β-actin images are from the same blot, while the HK2 images are from an upper blot of the PD-L1.

**
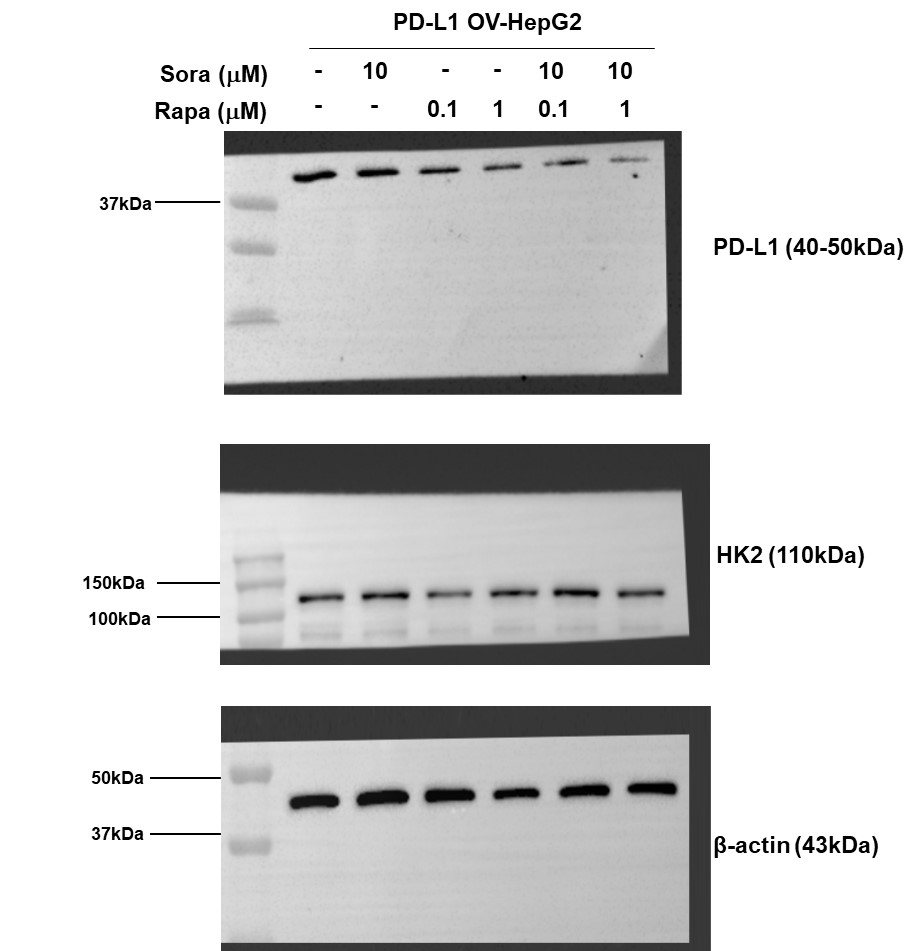
**

**Supplementary Figure S10.** Whole blot images of Fig 4c. HK2 images are from an upper blot of the PD-L1.


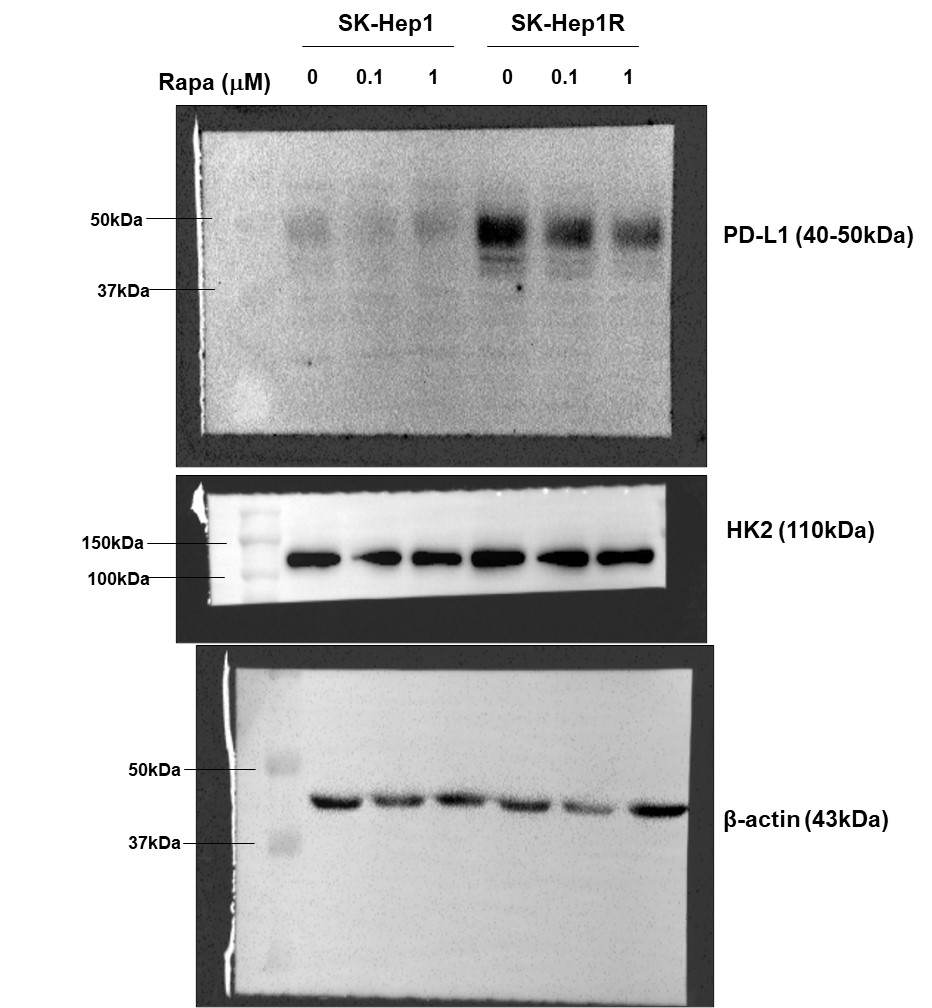


**Supplementary Figure S11.** Whole blot images of Fig 4e. The PD-L1 and β-actin images are from the same blot, while the HK2 images are from an upper blot of the PD-L1.

**Supplementary Table S1. Characteristics of patients with HCC in tissue microarray.**

| **Patients (n)** |  | **n** |  |
| --- | --- | --- | --- |
|  |  | 24 |  |
| **Age (years)** |  | **Median (range)** |  |
|  |  | 49.5 (36–71) |  |
| **Sex (n)** |  | **n** | **%** |
|  | **Male** | 20 | 83.33 |
|  | **Female** | 4 | 16.67 |
| **TNM stage of primary tumor (n)** |  | **n** | **%** |
|  | II | 6 | 25 |
|  | IIIA | 16 | 66.66 |
|  | IIIB | 2 | 8.33 |
